# Evaluation of the Catalytic Effect of Metal Additives on the Performance of a Combined Battery and Electrolyzer System

**DOI:** 10.1021/acsaem.4c02648

**Published:** 2025-01-08

**Authors:** Elizabeth Ashton, Matthew Brenton, Jonathan G. Wilson, John P. Barton, Richard Wilson, Danielle Strickland, Simon. A. Kondrat, Nicolas. Clement, John. Wertz, Jibo. Zhang

**Affiliations:** † CREST, Wolfson School of Mechanical, Electrical and Manufacturing Engineering, Loughborough University, Loughborough LE11 3TU, U.K.; ‡ Department of Chemistry, School of Science, Loughborough University, Loughborough LE11 3TU, U.K.; § Hollingsworth and Vose, Groton, Massachusetts 01450, United States

**Keywords:** Green Hydrogen, Hydrogen production, Electrolysis, Battolysers, Battery-Electrolyzers, Catalysts, Hydrogen evolution reaction

## Abstract

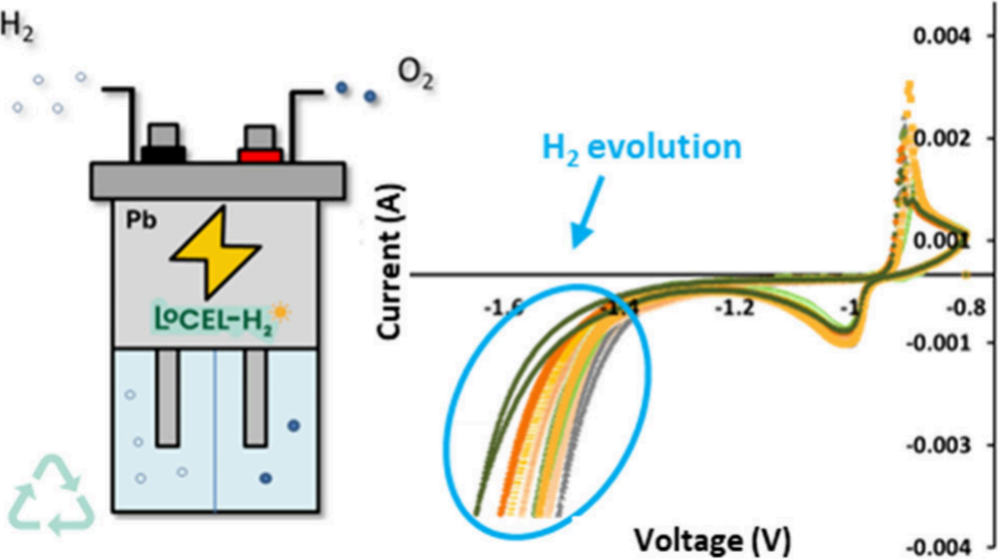

A low-cost method of green hydrogen production via the modification of a lead acid battery has been achieved, resulting in a hydrogen flow rate of 5.3 L min^–1^ from a 20-cell string. The electrochemical behavior and catalytic effect of various metal additives on the hydrogen evolution reaction (HER) was evaluated using cyclic voltammetry. Nickel, cobalt, antimony, manganese, and iron were investigated, with 66 ppm nickel achieving a 75% increase in hydrogen produced from a modified lead acid battery. Design of Experiments (DOE) employing a simple centroid design model to analyze the combined additive effects of nickel, cobalt, and antimony was performed to evaluate the effect on the HER. A combination of Ni:Co:Sb in the ratio 66:17:17 ppm achieved the greatest end voltage shift of the HER from −1.65 to −1.50 V; however, no increase in hydrogen yield was observed in comparison to 66 ppm of nickel when added to a full-scale cell. Gas chromatography using a thermal conductive detector and a sulfur chemiluminescence detector were used to measure the purity of hydrogen obtained from a string of 20 battery electrolyzer cells connected in series. 99% purity hydrogen gas was obtained from the battery electrolyzer cells, with H_2_S impurities below the limit of detection (0.221 ppm).

## Introduction

Grid-scale energy storage is required to compensate for the intermittent nature of renewable energy. Lead-acid batteries are commonly utilized in backup power applications and grid arbitrage systems due to their rapid response times.^1^ However, their market share is declining in favor of lithium-ion batteries, which offer higher energy densities. Despite this advantage, lithium-ion technology presents challenges, including an underdeveloped recycling market and significant safety concerns. In contrast, lead-acid batteries are highly recyclable, with 99% of their materials recoverable, offering a significant advantage for stationary storage applications.^2,3^

A variety of metallic impurities inherent to lead-acid batteries can catalyze undesired reactions, leading to an increased production of hydrogen (H_2_) and oxygen (O_2_) gases within the cell. Impurities in the electrolyte solution can have a significant impact on the battery performance. The maximum allowable concentrations of impurities are therefore strictly regulated to ensure safe and efficient battery operation.^4,5^

The gassing of a lead acid battery cell must be minimized to reduce the production of an explosive mixture of hydrogen and oxygen gas. This increased gassing within lead acid batteries is usually undesirable due to the increased safety risks as well as decreased performance and durability of the cell. However, modification of a lead acid battery to allow complete separation of the hydrogen and oxygen gas produced at each electrode can increase the safety of the cell and provide a method of green hydrogen production using abundant and low-cost materials.^6^

Hydrogen production via water electrolysis has received significant attention as a clean and sustainable energy solution. Advances in electrocatalyst design, particularly for water electrolysis, have focused on improving the efficiency and cost-effectiveness of hydrogen production. Studies have explored the development of nonprecious metal catalysts, which can replace expensive materials like platinum and iridium in both alkaline and proton exchange membrane (PEM) electrolysis systems.^7^ Innovations in electrocatalyst materials, including transition-metal-based catalysts, have further enhanced the performance and durability of these systems.^8^ These improvements have been essential for advancing the commercial viability of green hydrogen production.^9^

Our research enabled the design and development of an integrated battery and electrolyzer system, facilitating controlled overcharging to yield pure hydrogen gas. By integrating battery and electrolyzer functions into a single unit and utilizing common metal additives to enhance hydrogen production, our system overcomes the limitations identified in existing technologies, providing a more efficient, sustainable, and economically viable solution for grid-scale energy storage.

**Figure 1:**
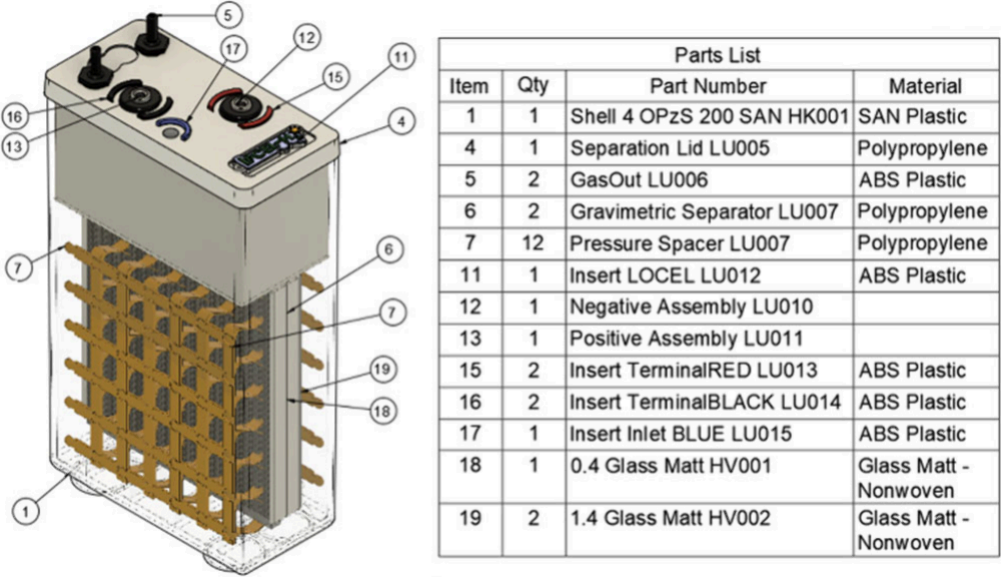
Complete CAD design drawing of the battery-electrolyzer cell.

The hydrogen gas obtained from the combined battery and electrolyzer can be used for further energy storage. As a result, the previously undesired gassing of the lead acid battery can instead allow operation as an electrolyzer. The unit retains its existing battery functionality, resulting in a single device known as a battery-electrolyzer. The design of our system is such that complete separation of the oxygen and hydrogen gas can be achieved by the introduction of a separator to the modified lead acid battery as displayed in Figure 1.

A specifically designed 3D printed separator developed at Loughborough University was added between the negative active mass (NAM) and positive active mass (PAM) to ensure complete separation of the hydrogen and oxygen gas produced at each electrode, respectively. Additionally, the cell lid was purposely designed and manufactured to ensure no crossover of the gases and safe and controlled release from the cell at ambient pressure.

The cells can be combined in parallel or in series to achieve the required power output and can be scaled up for a variety of energy usages. As a result, the combined battery-electrolyzer can offer a low-cost solution for coupling with poor load factor energy production such as that seen with renewable energy. The cells can be operated as a standard lead acid battery, where the cell is charged during renewable energy production and discharged to meet load demands.^6^ During peak energy production, excess renewable energy can continue to be harnessed by the production of hydrogen via the overcharging of the cells. If an electrolysis voltage is applied to a fully charged cell, hydrogen is produced and collected for further chemical energy storage.

No expensive catalysis, such as iridium or platinum used in standard electrolyzer stacks, is required by using well-known lead acid electrochemistry and metal additives. The addition of certain additives can increase the amount of hydrogen produced from the combined lead battery electrolyzer cells.^12^

Understanding the HER that occurs at the NAM and the oxygen evolution reaction (OER) at the positive active mass (PAM) during the charging process, as well as the catalytic effects of different metals, is required to develop strategies to ensure maximum performance and safe operation of the cells.

The half-reaction for hydrogen evolution from electrolysis during charging of the NAM is
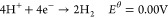
1while the oxygen evolution that occurs at the positive active mass (PAM) is
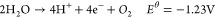
2

The overall reaction for the charge and discharge of a lead acid battery is
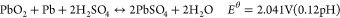
3

During discharge of the lead acid battery electrolyzer, oxidation of the lead (Pb) NAM occurs forming lead sulfate (PbSO_4_) by the forward reaction of the following reversable pathway:
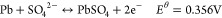
4while reduction of the PAM lead oxide (PbO_2_) also forms lead sulfate during discharge via the following forward reaction route:
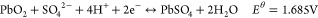
5

During charging of a battery electrolyzer cell, the NAM is reduced back to Pb, while the PAM is oxidized back to PbO_2_. If an electrolysis voltage is applied to the cell, then during recharging inefficiencies of conversion produce H_2_ and O_2_ via splitting of the water in the electrolyte solution.^12^

Previous research has focused on the development of flow batteries electrolyzers, including nickel flow batteries and soluble lead flow batteries.^10,13^ Unlike conventional batteries, where energy is stored within the electrodes, in flow batteries the energy is stored in the electrolyte solutions. As a result, external pumps are required to pump the electrolyte solutions through the cell stack when the battery is charged or discharged. Flow batteries require complex infrastructure with external pumps and separate electrolyte storage, increasing system complexity and cost.^6,10,11^ However, with the modification of a standard lead acid battery to produce the battery electrolyzer, no external electrolyte pumps are required.

As displayed in Figure 1 the cell is one combined unit. The electrodes used are identical to that of standard lead acid batteries, with an electrolyte solution of 4 M sulfuric acid solution. Recyclable plastics, such as those commonly used for lead acid batteries, are used for lid manufacture. Spent cells can therefore be recycled using the already existing infrastructure for recycling lead acid batteries.^14^

**Figure 2:**
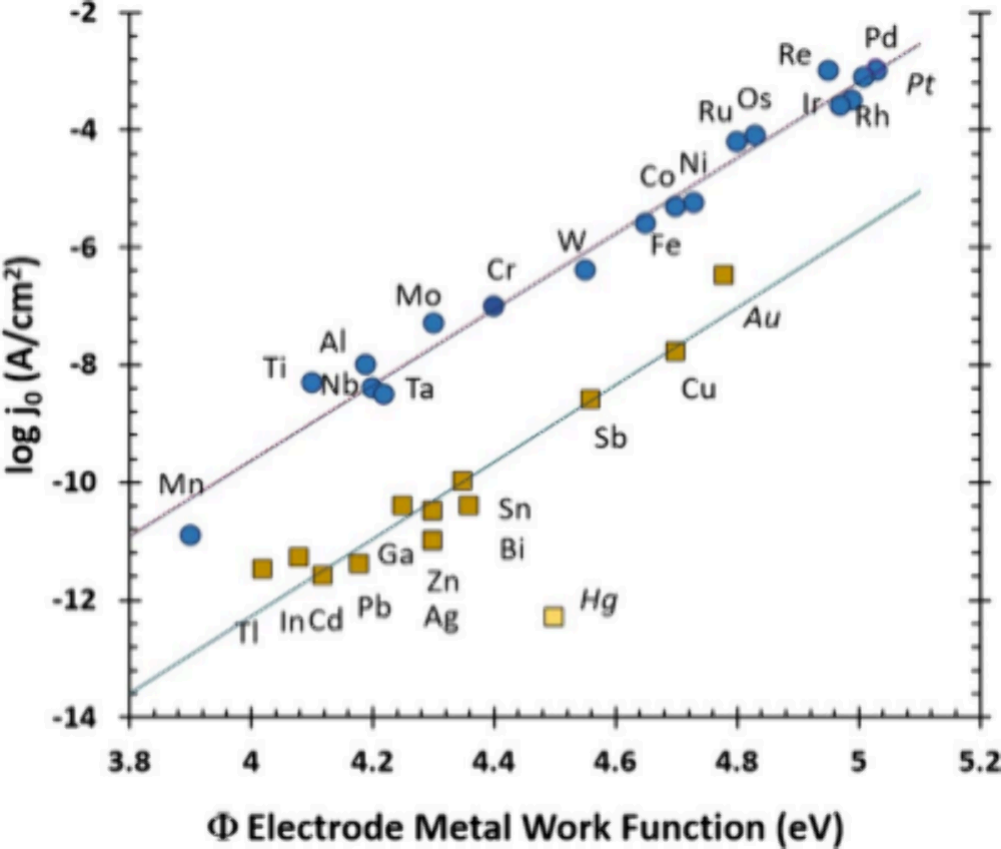
An Eyring plot comparison of 31 metal electrodes with *d* valence electrons (blue) and *sp* valence electrons (yellow).^5,16^

As long as the water content of the sulfuric acid electrolyte solution is maintained at an optimal density for operation of the lead acid battery (using an automated top-up valve), then the cell will continue to produce clean hydrogen via electrolysis during overcharging.^15^ The hydrogen yield obtained from the cell when operated as an electrolyzer can be increased further by the addition of metal additives. The metal additives were selected from the 31 polycrystalline metal elements used as electrodes in sulfuric acid electrolyte that were displayed in the Eyring plot produced by Trasatti in 1972 (Figure 2).^5^ The graph demonstrates the effect of the hydrogen evolution reaction (HER) on metal electrode surfaces of metals with d valence electrons (top) and sp valence electrons (lower line).

Platinum (Pt) and palladium (Pd) have the largest work function (Φ), respectively, and therefore greatest catalytic effect on the HER; however, as they are both rare and precious metals, they have a high cost associated so were not selected for additive testing to the battery electrolyzer cells. Additionally, the high catalytic effect of Pt and Pd inhibits and restricts lead battery recharging. Instead nickel, cobalt, and iron were selected due to their high work function.^16^ Manganese was selected as it has a low toxicity and low cost. Antimony was also selected, as it is present in some lead acid battery electrodes to provide strength.

This study therefore investigates the catalytic effects of nickel, cobalt, antimony, manganese, and iron on hydrogen and oxygen production within a lead-acid battery-electrolyzer system.

## Experimental Procedure

Cyclic voltammetry was performed using a 3-electrode Arbin Mstat 4 cells cycler/cv unit, including a rotary lead disk PINE electrode, to determine the electrochemical effect of the additives on the hydrogen evolution from 4 M sulfuric acid electrolyte solution. The manganese, nickel, cobalt, and antimony additives were tested in the concentration range of 1–100 ppm, while iron was tested in a concentration range of 150–1000 ppm. These concentration ranges were selected based on the maximum allowable concentration for metal impurities in lead acid batteries, as stated by D. Pavlov.^4^

The minimum allowable concentrations of various impurities are as follows:

1. below 1 ppm for tellurium, antimony, arsenic, cobalt, and nickel.
2. below 3 ppm for manganese.
3. below 160 ppm for iron.
4. below 500 ppm for aluminum, bismuth, cerium, chromium, copper, molybdenum, silver, and vanadium.
5. below 5000 ppm for barium, cadmium, calcium, chlorine, lithium, mercury, phosphorus, tin, and zinc.

It was therefore predicted that gassing would occur above these limits. The additives were introduced to the electrolyte solution by the addition of the metal sulfate (nickel sulfate hexahydrate, iron sulfate heptahydrate, cobalt sulfate heptahydrate, manganese sulfate monohydrate, and antimony sulfate) all purchased from Sigma-Aldrich ≥99.99% trace metals.

An anodic voltage scan started at +0.800 V using a mercury-mercurous sulfate Hg/Hg_2_SO_4_ reference electrode and counter electrode made from pure lead of minimum 99.99% purity. The rotating working lead electrode voltage was increased to a cell current of at least 5.5 mA, which resulted in a disc voltage of approximately +1.60 V. At 5.5 mA, the sweep direction was reversed and returned the disc potential to the initial value of +0.800 V. A cathodic scan was performed similarly between −0.800 and 1.60 V. This experimental design followed the industry-standard BCIS-03A version 2015 Battery Council International procedure for lead acid batteries.^17^

**Figure 3:**
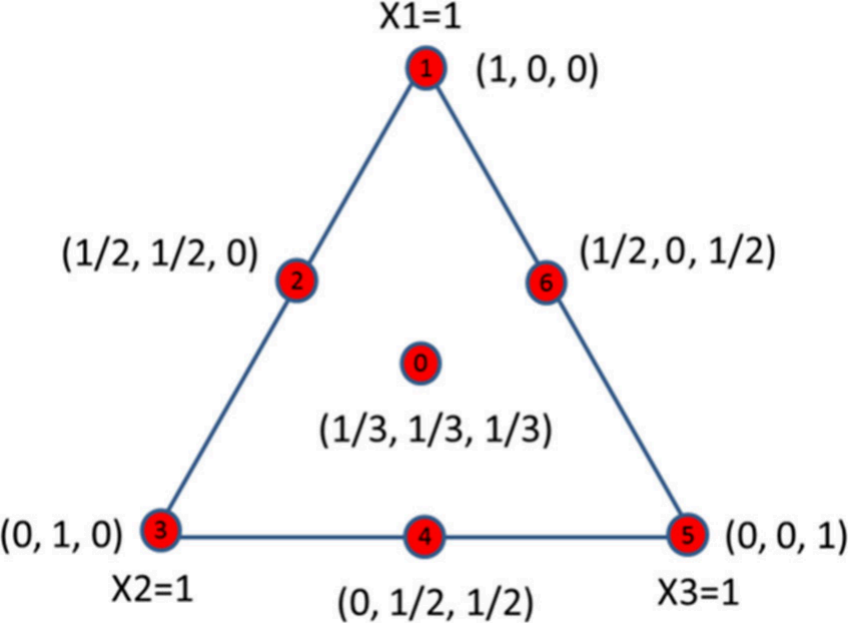
A schematic detailing the simple centroid design model used for the design of experiments and created using MODDE software.

A Design of Experiments (DOE) was executed using the MODDE software, employing a simple centroid design model to analyze the combined additive effects of nickel, cobalt, and antimony, as displayed in Figure 3.

Using the simple centroid design model, a total concentration of 100 ppm of combined additives was tested, via the ratios listed in Table 1.^18^

**Table 1:** Ratio of Ni:Co:Sb additives to 4 M sulfuric acid electrolyte solution for cyclic voltammetry measurements, including experiment number and run order determined from the design of experiment model

Exp No.	Run Order	Ni	Co	Sb
1	11	1	0	0
2	5	0	1	0
3	4	0	0	1
4	2	0.5	0.5	0
5	12	0.5	0	0.5
6	10	0	0.5	0.5
7	8	0.67	0.17	0.17
8	3	0.17	0.67	0.17
9	6	0.17	0.17	0.67
10	1	0.33	0.33	0.33
11	7	0.33	0.33	0.33
12	9	0.33	0.33	0.33

To evaluate the catalytic effect of the additives on a full-scale cell, further additive testing was conducted on an unmodified lead acid battery cell and also a modified battery-electrolyzer cell.

Electrolysis of the unmodified Hoppecke 4 OPzS solar power 280 cell was performed at a voltage of 2.9 V and current of 9.1 A, before and after addition of 250 ppm iron sulfate, 1–67 ppm nickel, and a combination of Ni:Co:Sb in the ratio 67:17:17 ppm. Hydrogen and oxygen gas was collected using the water displacement method to calculate the rate of gas produced with and without the additives.^19^ Electrolysis of the battery electrolyzer cell was also performed with the same additives, voltage, and current range. However, additional testing at higher power (3 V and 20 A) was also performed to further evaluate the effect of the additives and the overall performance of the battery electrolyzer cell.

As water was lost from the electrolyte solution during electrolysis, a standard lead acid battery water top-up valve was incorporated into the string of battery electrolyzer cells to ensure the electrolyte level was maintained at the correct height, concentration, and density. Each cell in the string was connected by 6 mm tubing to ensure a hydrostatic lock between the cells. A manual water top up was performed when a single cell was operated during electrolysis testing.

Electrical impedance spectroscopy (EIS) was performed to examine the impedance response of the combined battery and electrolyzer cells in different frequency ranges. This involved applying a small alternating current (AC) signal to the battery and measuring the resulting voltage. The parameters used for EIS were an initial frequency of 9400 Hz and a final frequency of 0.1 Hz with 10 mV perturbation at no load.

### Cell Performance and Durability

To allow automation of the charging, electrolyzing, and discharging of the cells, Chroma programmable hardware was integrated with LabVIEW to create a program to test cycle the cells. Details of the power supply and load bank used are displayed in Table 2.

**Table 2:** Details and specification of the Chroma programmable hardware used for cycling of the battery electrolyzer cells

Name	Description	Spec
CHR62150H-450	3-Phase DC Power Supply	0–450 V 0–34 A
63200 Series	Load bank	0–80 V 0–300 A

LabVIEW was used to create a program that automated the control of the power supply units and load banks. This program allowed us to generate automated sequences for charging, discharging, electrolysis, and cell cycling. A LabVIEW script was created to control and read the voltage, current, and power of the power supplies and load banks. Additionally, the program ensured easy selection of the associated COM port for each device used for file control, with automated data saving.

A capacity test using a C24 rate was performed on the Hoppecke OPzS solar power 62 Ah plate pair with a discharge current of 2.55 A, while a C5 capacity test was performed using a C5 rate of discharge at 9.1 A. Cycling of the cells was performed at C5 using a charge voltage of 2.4 V. Electrolysis was performed at 2.9 V also at 9.1 A. A protection voltage of 1.75 V was applied to the system to prevent voltage collapse of the cells during discharge.

### Gas Chromatography Analysis

Gas chromatography (GC) of a sample of hydrogen and oxygen gases obtained from the cells was collected and tested for impurities using two different methods of analysis. To determine the concentration of sulfur containing impurities, a sulfur chemiluminescence detector (SCD) using a CPSil-5 CB column with a helium flow gas was used. Additionally, a GC with thermal conductance detector (TCD) was used for analysis of oxygen and hydrogen crossover.

## Results and Discussion

### Cycling Voltammetry Using Nickel, Cobalt, Manganese, or Antimony Additives of Various Concentration to the Electrolyte Solution

**Figure 4:**
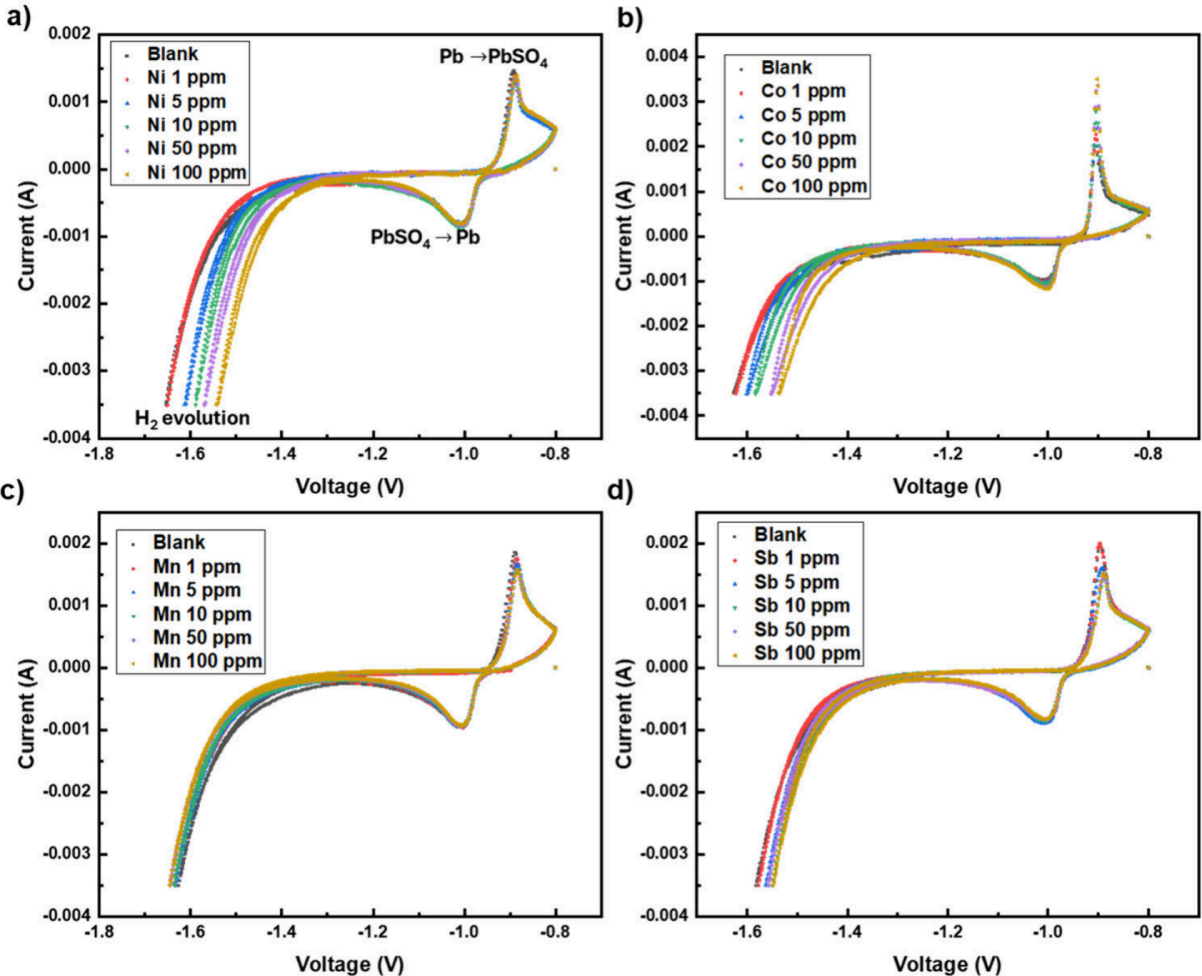
Cyclic voltammograms obtained from the cathodic scan of a lead electrode in 4 M sulfuric acid electrolyte solution, with a metal additive ((a) nickel, (B) cobalt, (C) manganese, and (D) antimony) in the concentration range of 1–100 ppm.

**Figure 5:**
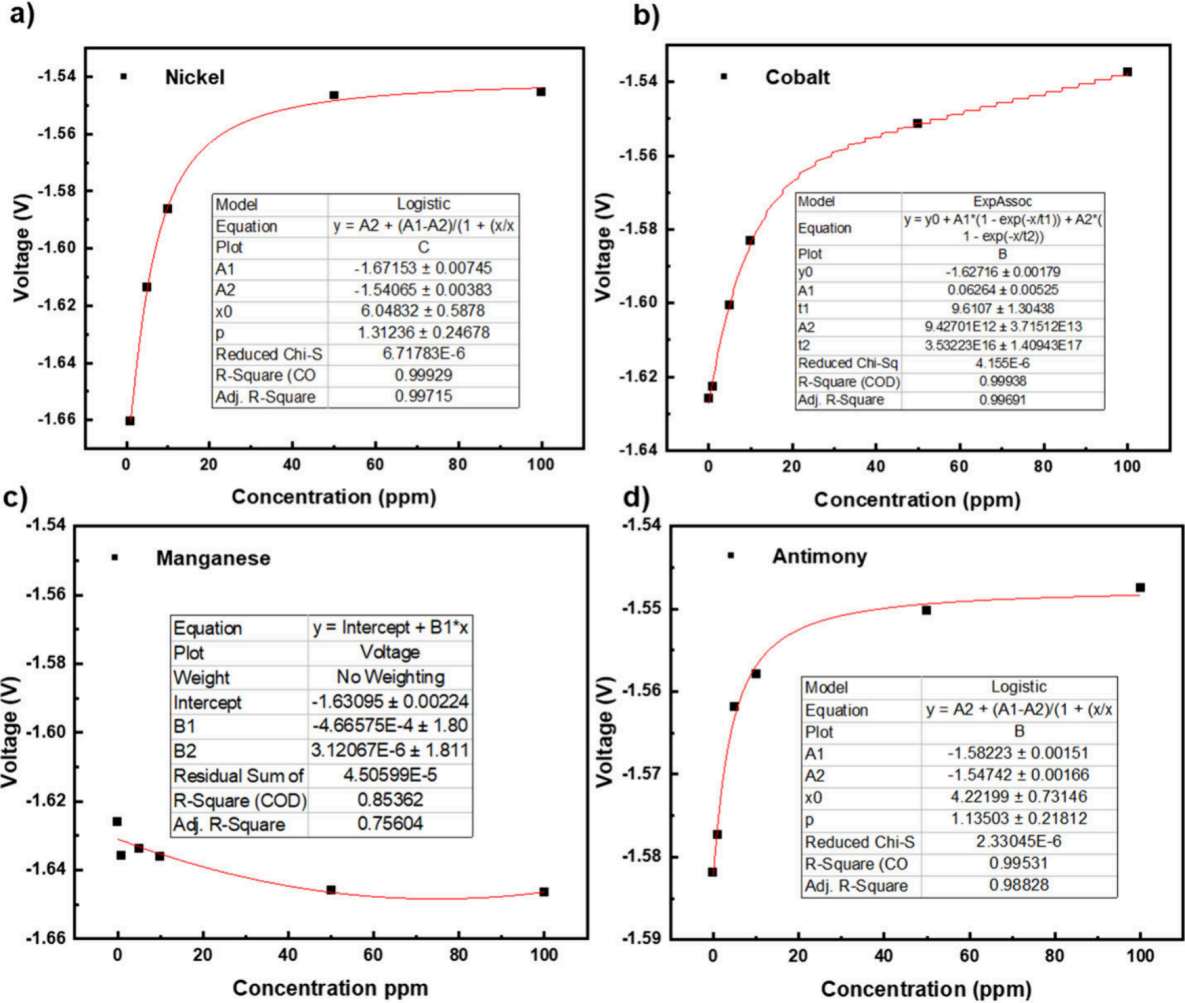
End voltage obtained from cyclic voltammetry experiments with varying concentration of additive (A) nickel, (B) cobalt, (C) manganese, and (D) antimony in 4 M sulfuric electrolyte solution, using lead as the working electrode.

The cyclic voltammograms obtained from the cathodic scan with a 1–100 ppm concentration range of nickel, cobalt, manganese, or antimony as an additive are displayed in Figure 4. Upon the addition of nickel in the form of nickel sulfate, the hydrogen evolution curve shifts to a less negative voltage, moving from −1.65 V to −1.54 V. This indicates that hydrogen evolution occurs at a less negative voltage due to the catalytic effect of nickel. At a low concentration of 1 ppm, there is no significant change in the end voltage of the hydrogen evolution section compared to a pure sulfuric acid electrolyte. However, as the nickel concentration increases to 100 ppm, the end voltage behavior follows a logistic model. The effect of additive concentration on the shift in end voltage of the hydrogen evolution curve of the cyclic voltammograms is displayed in Figure 5, for Ni, Co, Mn, and Sb.

Since manganese is a cheaper alternative to nickel, it was also tested as an additive to enhance hydrogen evolution during electrolysis in a combined battery and electrolyzer cell. However, no shift in the end voltage of the hydrogen evolution curve was observed across the concentration range. This is likely due to manganese’s lower metal work function (3.9 eV) and lower exchange current density compared to other metals like nickel, as shown in Figure 2.

When cobalt was used as an additive, a shift in the end voltage from −1.63 V to −1.54 V was observed. The overall voltage change of 0.09 V was slightly smaller than the 0.11 V shift seen with nickel, although both additives at 100 ppm resulted in a final voltage of −1.54 V.

Antimony was also tested as a potential catalyst. Antimony is commonly found in lead-acid battery electrode plates as an alloying agent, and small amounts may leach into the sulfuric acid electrolyte over time due to electrode degradation.^20^ Antimony’s catalytic properties make it unsuitable for use in maintenance-free batteries, as even small amounts (less than 1 ppm) can catalyze hydrogen gas formation. Concentrations of antimony between 1 and 100 ppm were tested, and the end voltage of the hydrogen evolution shifted slightly from −1.58 V to −1.55 V, a smaller change compared to nickel or cobalt. This reduced shift is likely due to differences in antimony’s outer electron orbital configuration (sp) compared to nickel and cobalt (d), which leads to a different reaction pathway. As shown in Figure 2, elements with sp orbital electrons have lower work functions, resulting in lower exchange current densities.

Like nickel, the results for antimony also fit a logistic model, suggesting concentration-dependent catalytic activity. Beyond 30 ppm, further increases in concentration had minimal changes in the end voltage, indicating a saturation effect. This saturation suggests that the metals act as heterogeneous catalysts by plating onto the electrode surface, providing a lower-energy surface for hydrogen evolution. However, the electrode surface may become saturated, limiting the number of proton adsorption sites.

During electrolysis performed using a lead electrode and 4 M sulfuric acid solution, the addition of additives (cobalt, nickel, and antimony) reduces the voltage at which the HER occurs. This effect arises because these metals act as an HER catalyst, enhancing the kinetics of the reaction and thereby lowering the overpotential required for hydrogen evolution. These metals are known to catalyze the HER through the Volmer-Heyrovsky or Volmer–Tafel mechanisms:

- Volmer Step (Proton adsorption):
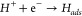
6
  Protons from the acidic solution are reduced and absorbed onto the electrode surface.
- Heyrovsky Step (Electrochemical desorption):
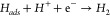
7

Molecular hydrogen forms when a proton and an electron combine with an adsorbed hydrogen atom.

Tafel Step (Chemical desorption):
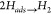
8

Hydrogen gas forms when two adsorbed hydrogen atoms recombine.^21,22^

The Butler–Volmer equation describes the relationship between the current density and overpotential for electrochemical reactions. In the presence of an additive, the exchange current density (*j*_0_) increases because the metal lowers the activation energy for HER. This enhancement reduces the required overpotential (η) for the reaction to proceed:
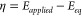
9

With nickel, cobalt, and antimony, the lower overpotential means hydrogen evolution starts at a lower voltage, indicating improved reaction kinetics.

**Figure 6:**
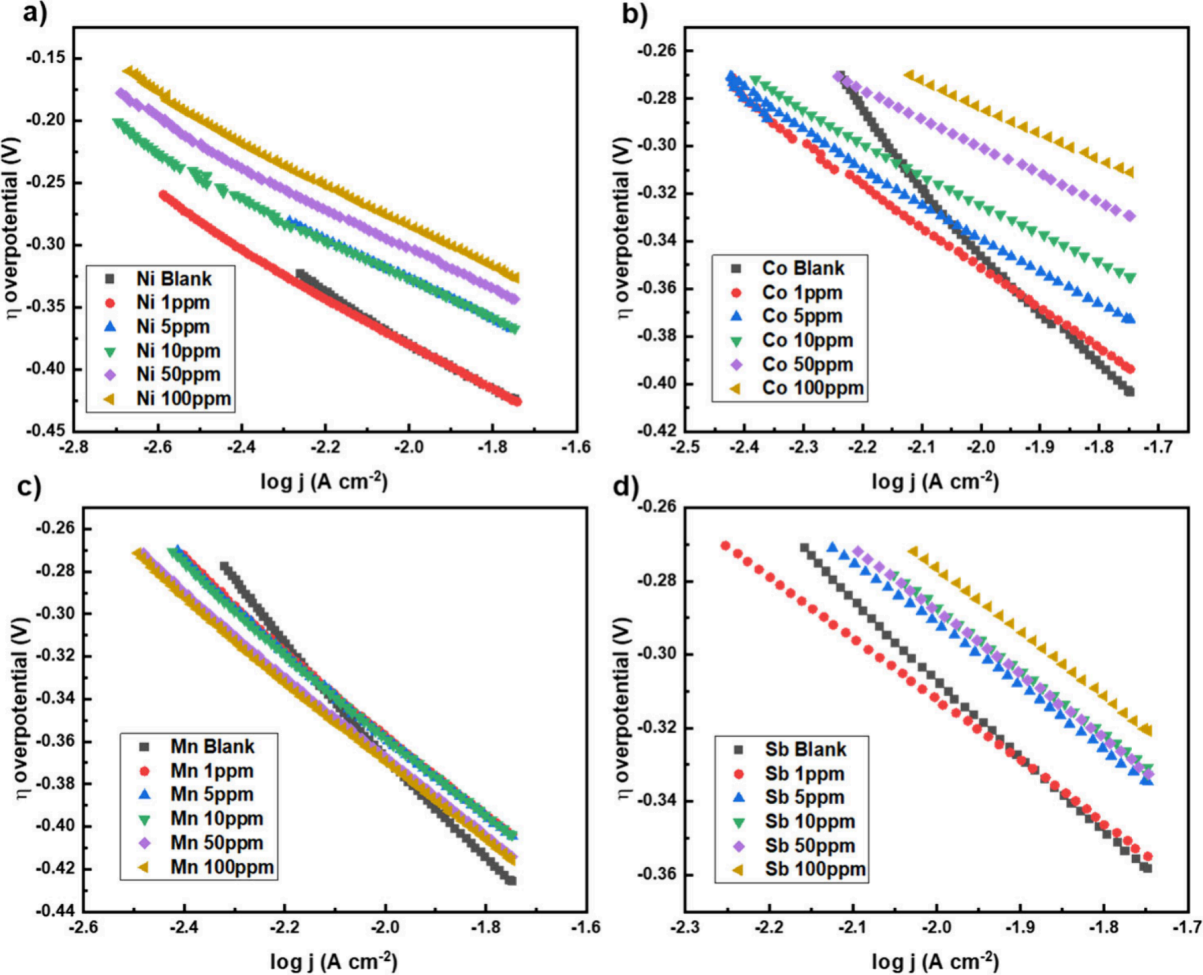
Tafel plots produced during electrolysis, when using a lead electrode (in a three-electrode system) and sulfuric acid electrolyte with addition of additive (A) nickel, (B) cobalt, (C) manganese, and (D) antimony.

Figure 6 displays the Tafel plots obtained from the hydrogen evolution that occurred when the overpotential was applied to the three-electrode system.

The Tafel equation is derived from the Butler–Volmer equation and relates the overpotential to the logarithm of the current density:
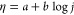
10where *b* is the Tafel slope. The slope provides insight into the rate-limiting step of the HER.

Enhanced kinetics of the HER is reflected in the plots via lower overpotentials and reduced Tafel slopes, indicating faster reaction rates and a shift in the rate-determining step. Although manganese did not reduce the voltage of the hydrogen evolution reaction, its catalytic effect can be seen by a reduction in Tafel slope with increasing concentration. Therefore, less overpotential is required to achieve the same increase in the reaction rate.

Adding these metal additives reduces the overpotential at the same current density for hydrogen evolution, which is highly beneficial for an electrolyzer because it improves energy efficiency. Lower overpotential means less voltage is needed to drive the reaction, reducing the overall energy consumption and operating costs. Additionally, it minimizes heat generation, improving the durability and lifespan of the battery electrolyzer. This increase in efficiency allows for the same hydrogen output with lower electrical input, making the process more cost-effective and sustainable.

**Figure 7:**
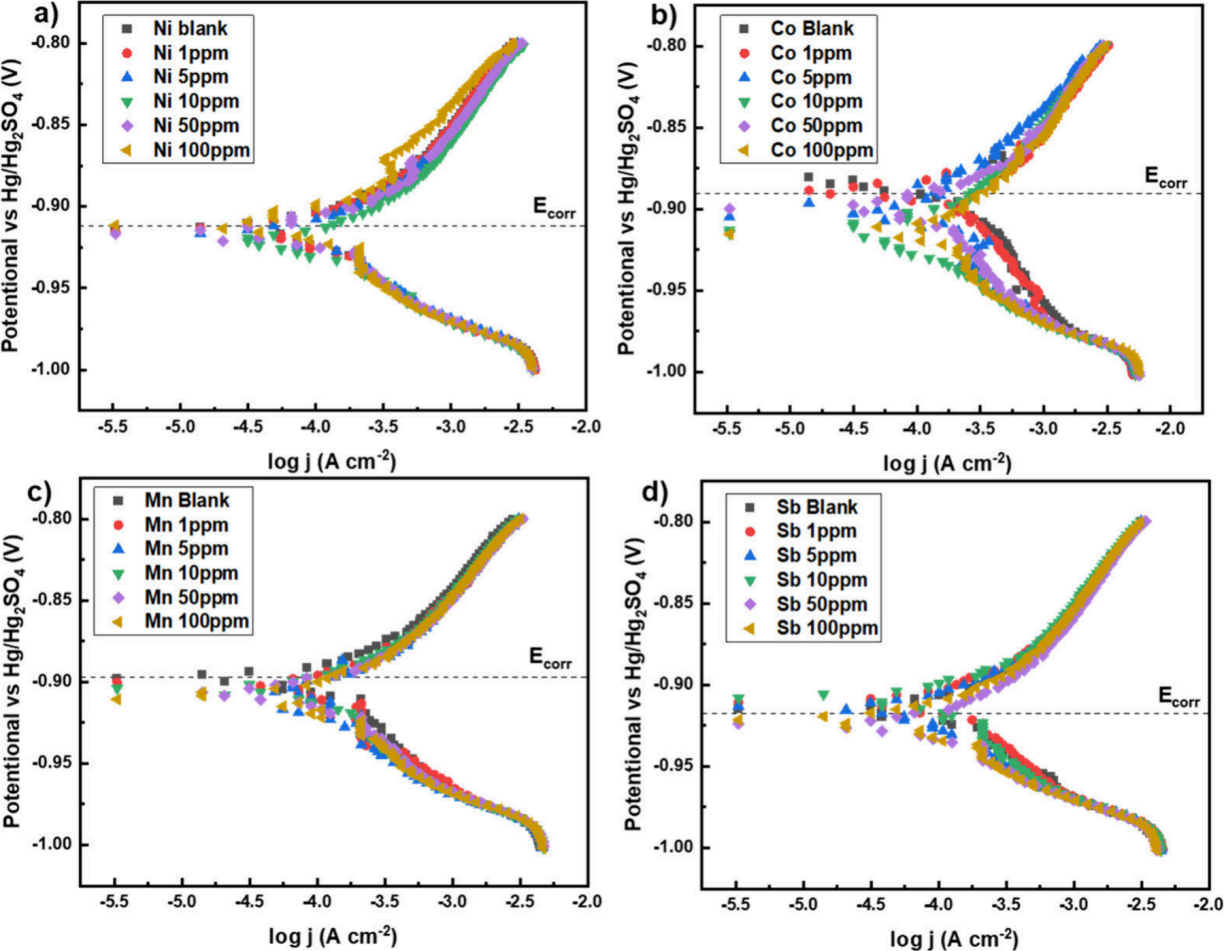
Tafel plots obtained from the cathodic scan during battery operation of a three-electrode system, with a lead working electrode, a Hg/Hg_2_SO_4_ reference electrode, and sulfuric acid electrolyte with addition of additive (A) nickel, (B) cobalt, (C) manganese, and (D) antimony.

A Tafel plot of the voltage range where the reduction and oxidation of the lead acid battery reaction mechanisms occur is shown in Figure 7.

The minimal impact of nickel addition (1–100 ppm) on the battery performance, as shown in the Tafel plot, is beneficial because it allows the lead-acid battery to function efficiently during charging and discharging without interference from the nickel catalyst. This selective effect means that nickel only enhances the HER during electrolysis, improving the system’s efficiency for hydrogen production, while preserving the battery’s normal electrochemical behavior. As a result, the cell can operate effectively both as an electrolyzer for hydrogen generation and as a reliable battery for energy storage.

Manganese, in contrast, showed a slight change in E_corr_ with increasing concentration. As manganese concentration increased from 0 to 100 ppm, the E_corr_ shifted to more negative values, from −0.898 V to −0.912 V. A more negative E_corr_ suggests that the manganese additive could lead to reduced stability over time.

The effect of antimony on E_corr_ was less consistent. At low concentrations (up to 10 ppm), E_corr_ shifted slightly toward less negative values, indicating improved stability and potential resistance to corrosion. However, at higher concentrations (50 and 100 ppm), E_corr_ shifted to more negative values, similar to the behavior seen with manganese. This suggests that while low concentrations of antimony may stabilize the lead electrode, higher concentrations may enhance reduction activity, which could decrease electrode durability.^20^

Cobalt exhibited the greatest effect on E_corr_, with a shift from approximately −0.890 V to −0.920 V as the concentration increased from 0 to 100 ppm. The more negative E_corr_ indicates that cobalt increases the reactivity of the lead electrode, potentially making it more prone to degradation.

In conclusion, the results obtained from adding nickel to the system demonstrated that the HER reaction can be catalyzed without affecting the battery performance. Increased degradation, particularly of the NAM, could occur on the addition of the other additives tested. Further research is required to assess the long-term impact of these additives on lead-acid battery performance, particularly at higher concentrations, where corrosion and electrode instability could become significant concerns.

### Cycling Voltammetry Using Iron Additive of Various Concentration to the Electrolyte Solution

**Figure 8:**
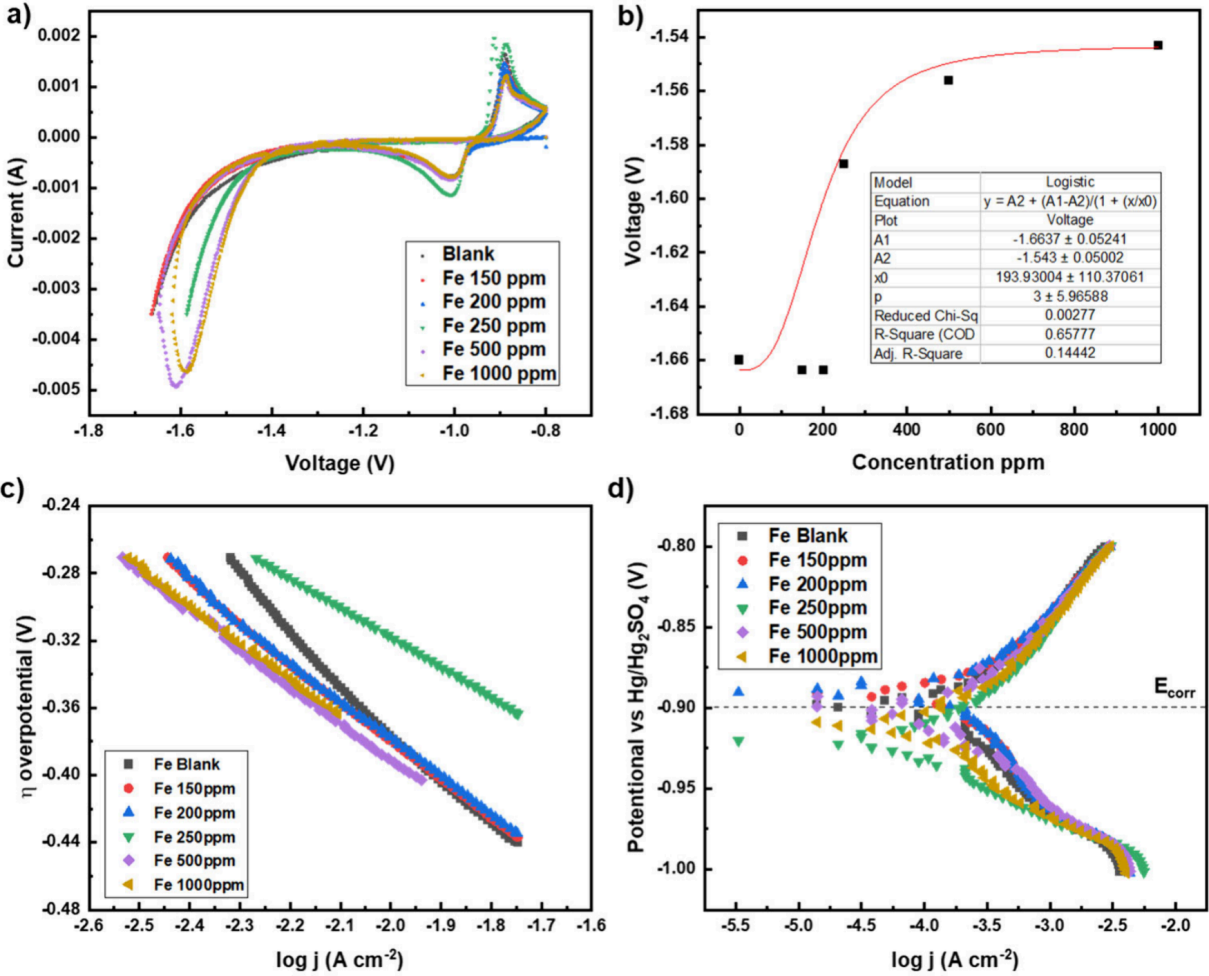
Results obtained from the cyclic voltammetry when iron (0–1000 ppm) is added to 4 M sulfuric acid electrolyte: (a) cyclic voltammogram, (b) shift in end voltage of the hydrogen evolution curve, (c) Tafel plot of the hydrogen evolution when applying over potential, (d) Tafel plot when operated as a battery.

Iron sulfate was selected as an additive due to its low cost, low toxicity, and high abundance; therefore, it is more economically viable and readily available in comparison to the other additives tested. The concentration range 150–1000 ppm was used, as the maximum allowable concentration of iron is 160 ppm, so concentrations above this range were predicted to cause increased gassing of the modified lead acid cell. The results obtained are displayed in Figure 8.

The voltammograms on addition of over 250 ppm iron additive demonstrate a distinctive “loop”, which is predicted to arise from the capacitance associated with electrolyte species adsorbed on the working electrode. The enclosed area within this loop appears to overlap with the current at which the hydrogen evolution reaction (HER) occurs. Again, the onset potential of the HER displays a positive shift with increasing iron concentrations until a voltage of −1.54 V is reached, as shown in Figure 8b. Therefore, the presence of looping at 250 ppm further confirms that this behavior may be indicative of a limitation in voltage capability and inability to surpass −1.54 V. The Tafel slope during electrolysis operation of the cell (Figure 8c) demonstrates the decrease in tafel slope when iron is added to the system, confirming the catalytic effect. During battery operation at lower concentrations (up to 200 ppm), E_corr_ shifted slightly toward less negative values, indicating an improved stability. However, at higher concentrations (over 250 ppm), E_corr_ shifted to more negative values, indicating increased degradation. Additionally iron cations can migrate between electrodes and result in self-discharge of lead acid batteries; therefore, high concentrations of iron impurities could be detrimental to the performance of the battery electrolyzer cell.^23^

### Design of Experiments (DOE) Cyclic Voltammetry Performed with Nickel, Cobalt, and Antimony Additives

**Figure 9:**
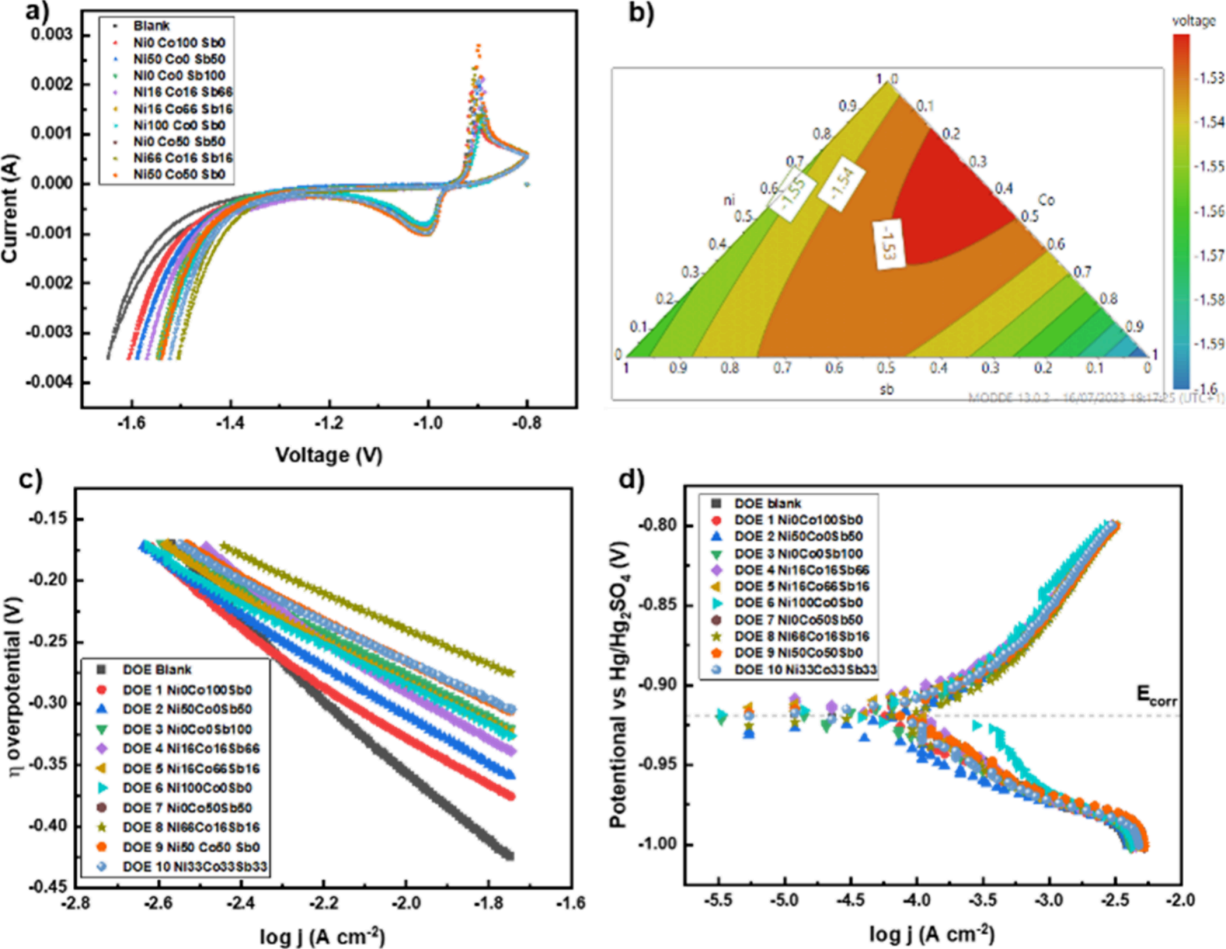
Results obtained from cyclic voltammetry measurements with a concentration of 100 ppm in varying ratio of additives nickel, cobalt, and antimony, using a DOE simple centroid design model; (a) cyclic voltammograms, (b) response contour plot of hydrogen evolution end voltage, (c) Tafel plot of hydrogen evolution curve during electrolysis, (d) Tafel plot when operating as a battery.

To determine the most effective additive and concentration and also explore any combination effects to determine if the voltage can be shifted beyond −1.54 V, a design of experiments (DOE) was performed using 100 ppm combined concentration of nickel, cobalt, and antimony in different ratios. The results obtained are displayed in Figure 9.

A composition consisting of 67 ppm of nickel (Ni), 17 ppm of cobalt (Co), and 17 ppm of antimony (Sb) achieved the greatest shift toward a less negative end voltage (−1.65 to −1.50 V), indicating a further 0.05 V increase compared to 100 ppm nickel alone. Figure 9b presents the response contour plot of the final cathodic voltages derived from the combined additive mixtures. Utilizing a combination of different metal additives successfully shifted the end voltage beyond −1.54 V to −1.50 V, suggesting the potential for hydrogen generation at higher voltages and increased yields.

On addition of additive, the Tafel slope obtained from the linear section of the hydrogen evolution curve (Figure 9c) was reduced, indicating a catalytic effect. As shown by the response contour plot, the additive ratio of Ni:Co:Sb 67:17:17 had the greatest reduction in the Tafel slope as well as reducing the overpotential required at a given current density. The Tafel plots obtained when operating the three-electrode system as a lead-acid battery (Figure 9d) demonstrate that the addition of this ratio of additives has minimal effect on oxidation and reduction of the NAM. The E_corr_ decreased slightly from −0.918 V to −0.926 V.

**Figure 10:**
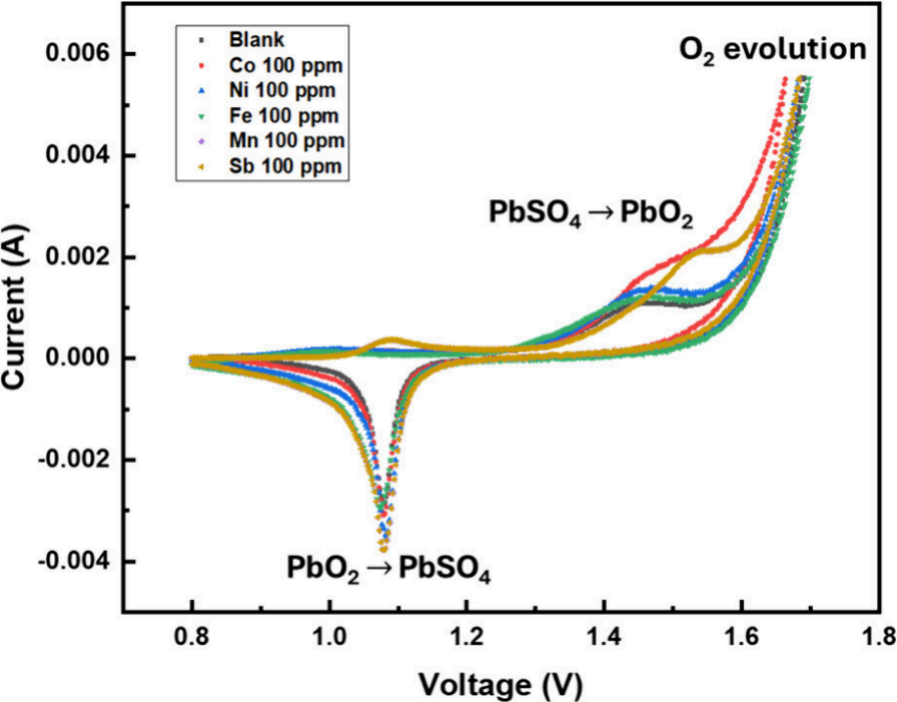
Oxygen evolution reaction that occurs when performing an anodic cyclic voltammetry scan in the positive direction, using nickel, cobalt, manganese, antimony, and iron additives to the 4 M sulfuric acid electrolyte solution.

In addition to the cathodic scan, an anodic scan in the positive direction was conducted for completion. Figure 10 displays the results obtained from the oxygen evolution reaction utilizing the lead working electrode, 4 M sulfuric acid electrolyte, and various additives, including nickel, cobalt, antimony, manganese, and iron.

The anodic scan shows an increase in current at approximately 1.4 V potential, which is associated with the onset of the oxygen evolution reaction (OER).^24^ The introduction of metal additives cobalt and antimony demonstrate an influence on the OER, while nickel, manganese, and iron did not have an effect on lowering the potential of the oxygen evolution reaction.^25^

### Electrolysis on a Full-Scale Battery-Electrolyzer Cell

**Figure 11:**
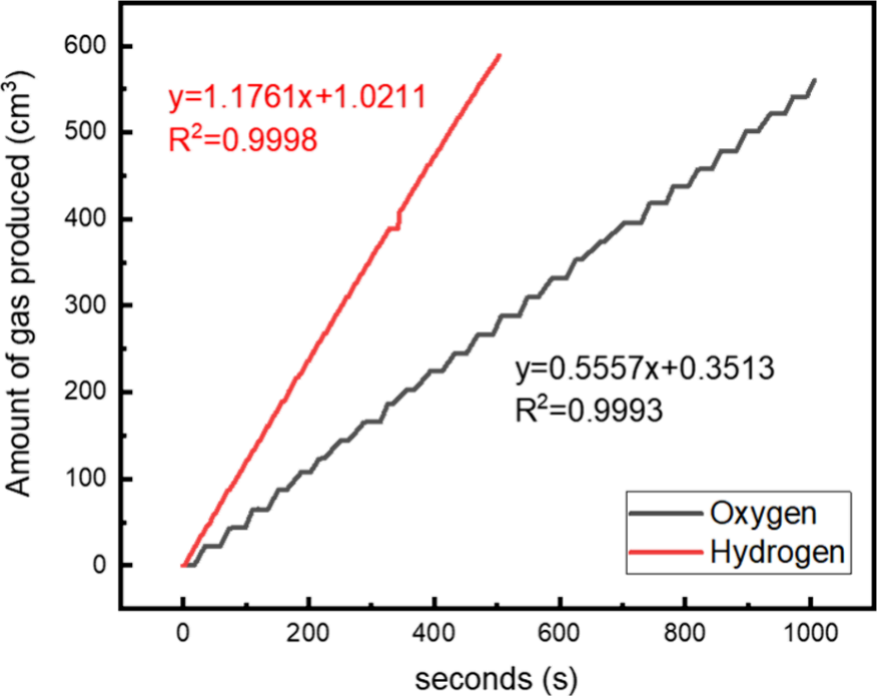
Oxygen and hydrogen measured gas collection from battery electrolyzer cell; hydrogen (red) and oxygen (black).

As previously mentioned, overcharging a lead-acid battery causes the generation of hydrogen gas at the cathode and oxygen gas at the anode. The released gases are typically vented and released from a lead acid battery to prevent an increase in the pressure and ensure safety. However, due to the modification of the cell via the cell lid design and separators used for the combined battery electrolyzer, the hydrogen gas could be collected from the negative electrode outlet and oxygen gas could be collected from the positive. Figure 11 shows the recorded hydrogen and oxygen evolutions from electrolysis of a full-scale battery electrolyzer cell.

The rate of hydrogen produced from the negative electrode was 1.18 ± 1.02 cm^3^ s^–1^, while the rate of oxygen produced from the positive electrode was 0.56 ± 0.35 cm^3^ s^–1^. The hydrogen rate of reaction was double that of the oxygen, which is expected due to the 2:1 ratio of hydrogen:oxygen in water (H_2_O). To ensure reliability and repeatability of the gas collection method, the electrolysis tests were repeated multiple times (results shown in the Supporting Information, Figure S1 and Figure S2). The average rate of oxygen produced was 0.546 ± 0.017 cm^3^ s^–1^ (32.76 ± 1.02 cm^3^ min^–1^), while the average hydrogen production rate was 1.207 ± 0.024 cm^3^ s^–1^ (72.42 ± 1.44 cm^3^ min^–1^), therefore confirming stoichiometric production of H_2_ and O_2_. These results were obtained from one cell using a maximum charge voltage of 2.9 V and a current of 9.1 A.

### Electrolysis on a String of 20 Battery Electrolyzer Cells

**Figure 12:**
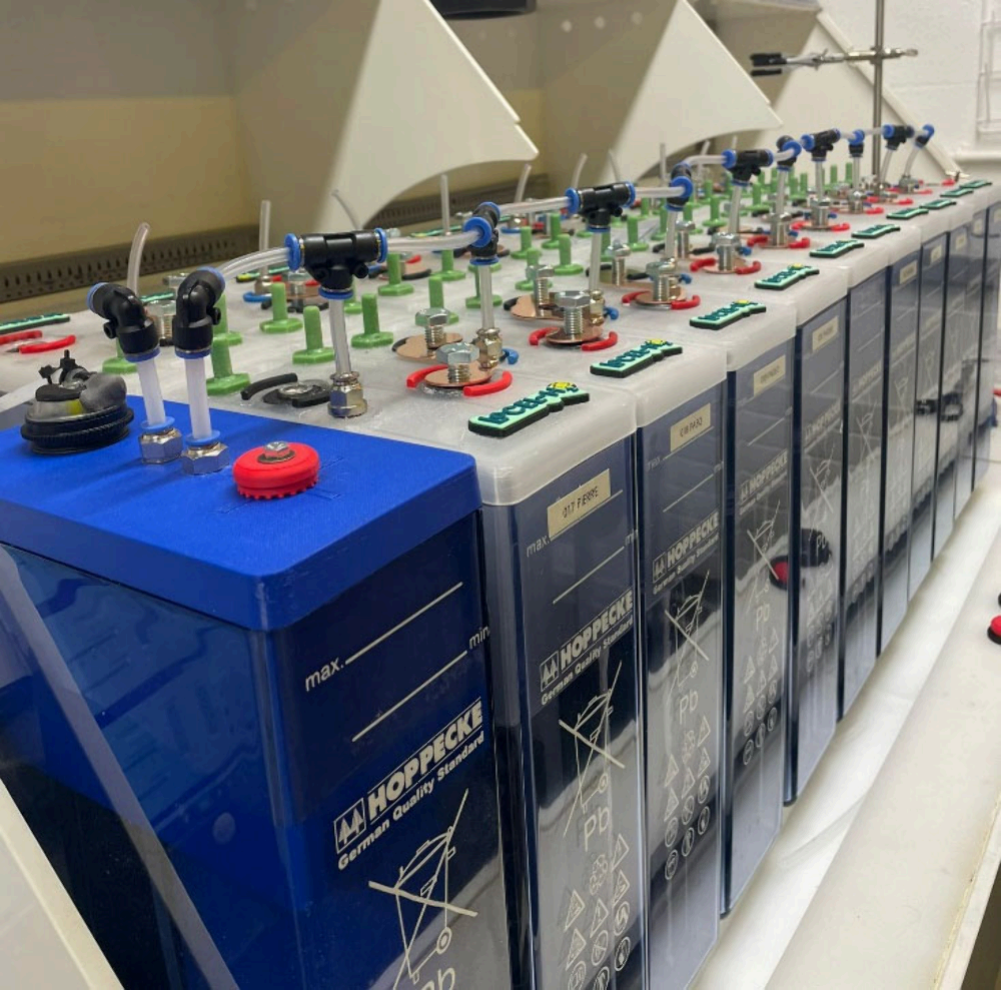
A photograph of 20 battery electrolyzer cells connected in series for hydrogen collection via electrolysis, also with a blue water top-up cell incorporated into the string.

A string of 20 battery electrolyzer cells, each with a capacity of 62 Ah, was connected in series as shown in Figure 12. Additionally, a blue water top-up cell was incorporated into the string, to automatically maintain the correct electrolyte level, concentration, and therefore density for battery operation.

**Figure 13:**
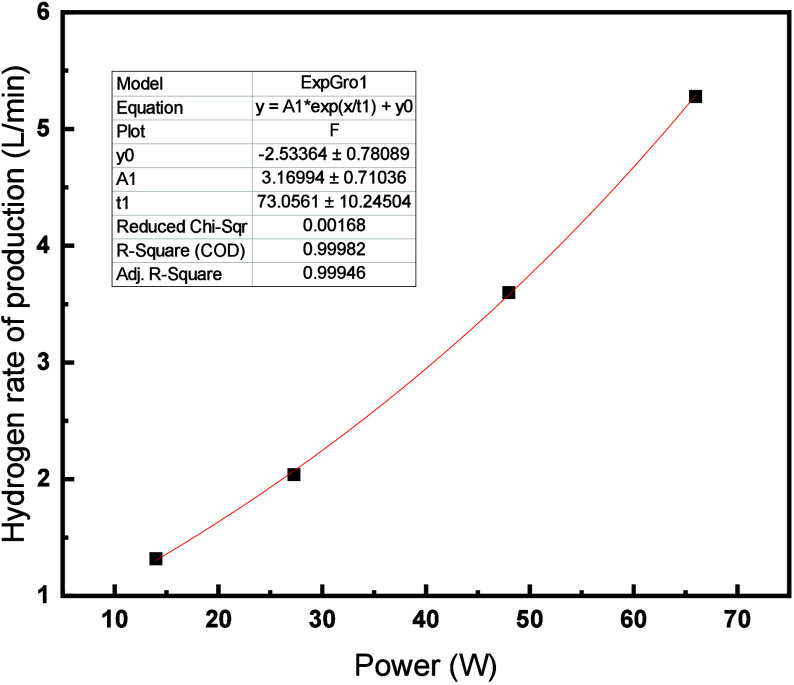
Hydrogen yield obtained from the electrolysis of a string of 20 battery electrolyzer cells at varying power.

The string of cells was electrolyzed at varying power, and the hydrogen yield was recorded, the results of which are displayed in Figure 13.

The current limit was varied to alter the rate of hydrogen generated by applying an electrolysis voltage of 2.9 V-3.1 V. The rate of hydrogen produced increased with increased overall power of the battery electrolyzer cells. When operating the string of 20 cells at 66 W the maximum rate of hydrogen produced was 5.3 L min^–1^.

### Electrolysis on a Full-Scale Battery-Electrolyzer Cell on Addition of Nickel, Cobalt, and Manganese Additives

**Figure 14:**
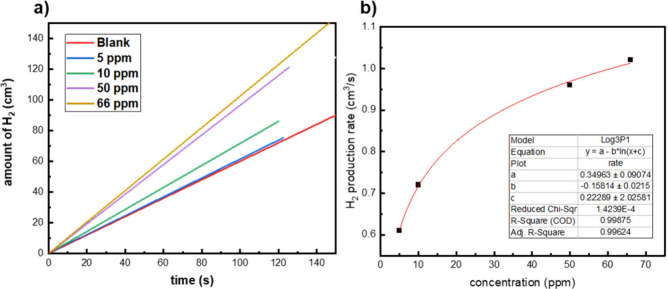
Yield of hydrogen obtained from an unmodified four-plate pair lead acid battery; (a) on addition of nickel additive in the concentration range of 0–66 ppm. (b) The rate of hydrogen produced.

To mimic the results obtained from the cyclic voltammetry testing, electrolysis of a lead acid battery with and without nickel additive was performed to allow comparison and determine whether additives to the electrolyte could increase the yield of hydrogen produced from the cell. The yield of hydrogen obtained on the addition of 0–66 ppm of nickel additive is displayed in Figure 14.

The amount of hydrogen produced when electrolyzing the unmodified cell at 2.9 V and 9.1 A increased with an increasing concentration of nickel, as expected. The rate of hydrogen production at each concentration of nickel is displayed in Figure 14b. As predicted from the small-scale testing of nickel additive to 4 M sulfuric acid electrolyte for cyclic voltammetry measurements, the increased concentration of nickel leads to a higher rate of hydrogen evolution due to the lowering of the voltage at which the HER occurs. It is predicted that at higher concentrations of 100 ppm, the rate of HER will begin to plateau.

**Figure 15:**
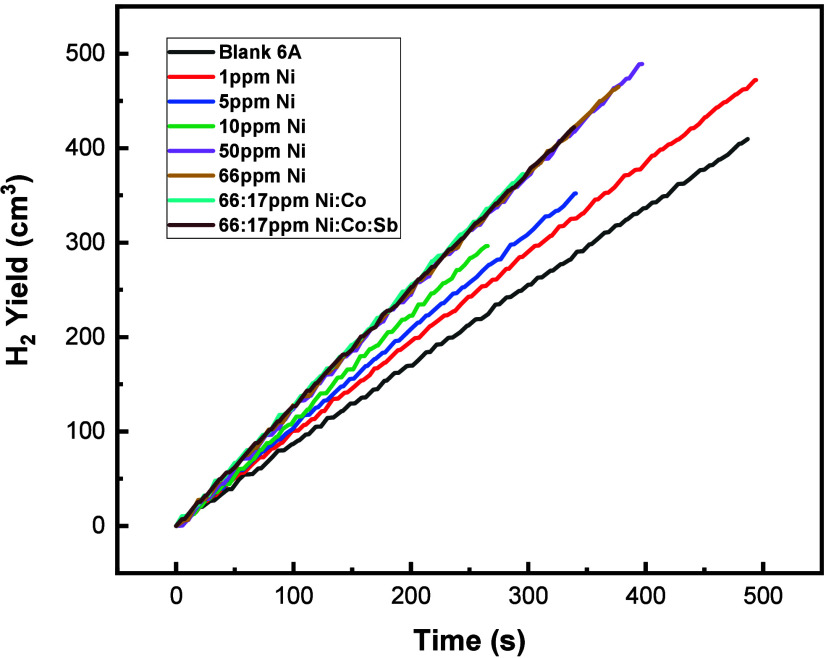
Hydrogen yield obtained from electrolysis of a battery electrolyzer cell on the addition of nickel additive at varying concentration (0–66 ppm), with the addition of Co and Sb in the ratio of Ni:Co:Sb 66:17:17 ppm.

Nickel additive was also added to a modified battery electrolyzer cell, followed by the addition of Co and Sb to recreate the final results obtained from the DOE performed during cyclic voltammetry testing. As the additive combination of Ni:Co:Sb 66:17:17 ppm achieved the greatest shift of the HER end voltage, the same ratio of additives was applied to the battery electrolyzer cell. The results obtained are displayed in Figure 15.

**Figure 16:**
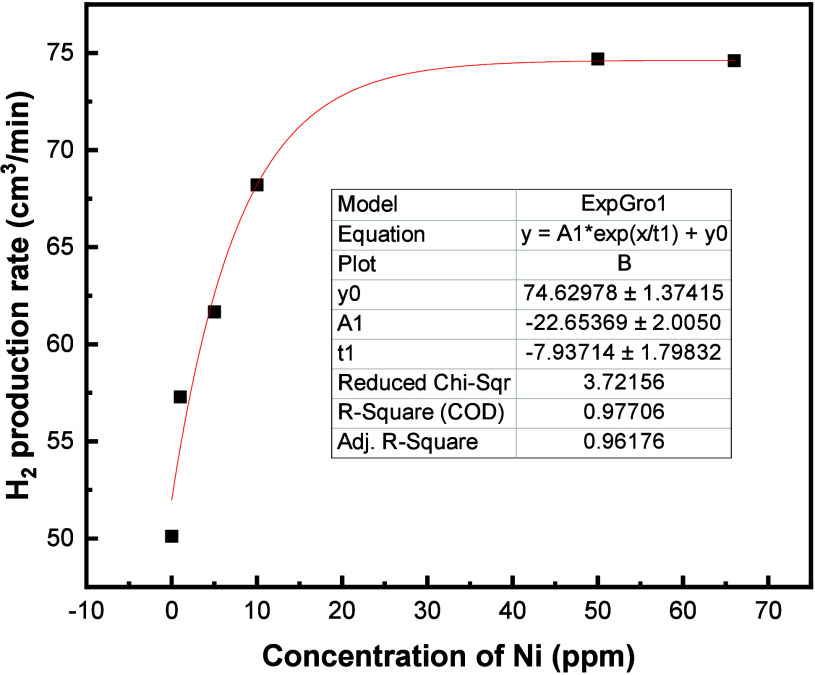
Rate of hydrogen produced during electrolysis of battery electrolyzer cell on addition of nickel additive.

As expected, the amount of hydrogen produced increased with an increasing concentration of Ni additive. However, no increase in rate of hydrogen occurred when Co and Sb were added to the 66 ppm of Ni, suggesting that the electrode surface was already saturated with metal additive; therefore, Co and Sb provided no further catalytic effect, despite the increased shift of the final electrolysis voltage obtained when performing DOE during cyclic voltammetry testing. The relationship between the rate of hydrogen produced during electrolysis and concentration of nickel added is displayed in Figure 16.

Plateau of the rate of hydrogen produced on the addition of nickel occurs past 50 ppm. Therefore, no further increase of hydrogen was obtained on addition of 17 ppm of Co and 17 ppm of Sb on a full-scale cell.

No catalytic effect was observed when the battery electrolyzer cell was electrolyzed at higher power (3.2 V and 20 A). This is likely due to the activation energy of HER relation being exceeding the increased energy supplied by the higher electrolysis voltage at 3.1 V and higher power of 20 A. Therefore, additives are not required if operating the battery electrolyzer cells at high power.

### Electrolysis on a Full-Scale Battery-Electrolyzer Cell on Addition of Iron Additive

**Figure 17:**
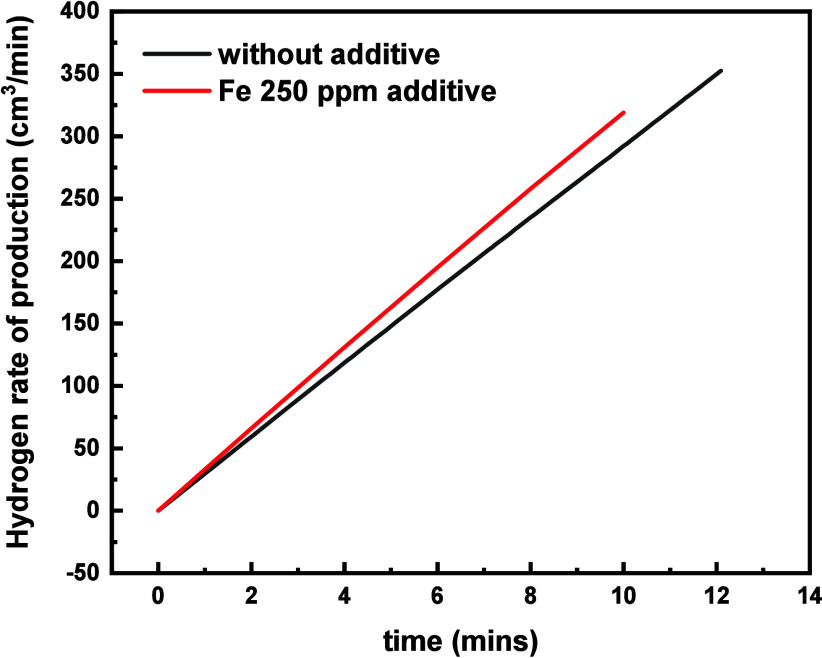
Hydrogen yield obtained from the addition of 250 ppm of iron to a battery electrolyzer cell.

Due to the low cost and high abundance of the material, iron sulfate was selected for electrolysis testing in electrolyte solution of the battery electrolyzer. An increased gas yield of 10.3% was observed on addition of the iron sulfate at a concentration of 250 ppm of iron in the sulfuric acid electrolyte solution (Figure 17).

The rate of hydrogen production at a constant current of 9.1 A without the iron additive was 29 cm^3^ min^–1^ and increased to 32 cm^3^ min^–1^ with additive, therefore concluding that the iron additive is a favorable and cost-effective option for the combined battery and electrolyzer system. However, at higher concentrations accelerated self-discharge may occur.^25^ When electrolyzing at 5 A the percentage increase of hydrogen rate of production on addition of 250 ppm of iron was 27%, and it dropped to only 3% increase at 15 A. Therefore, iron additive is beneficial only on the HER when operating at a lower power.

### Electrical Impedance Spectroscopy

**Figure 18:**
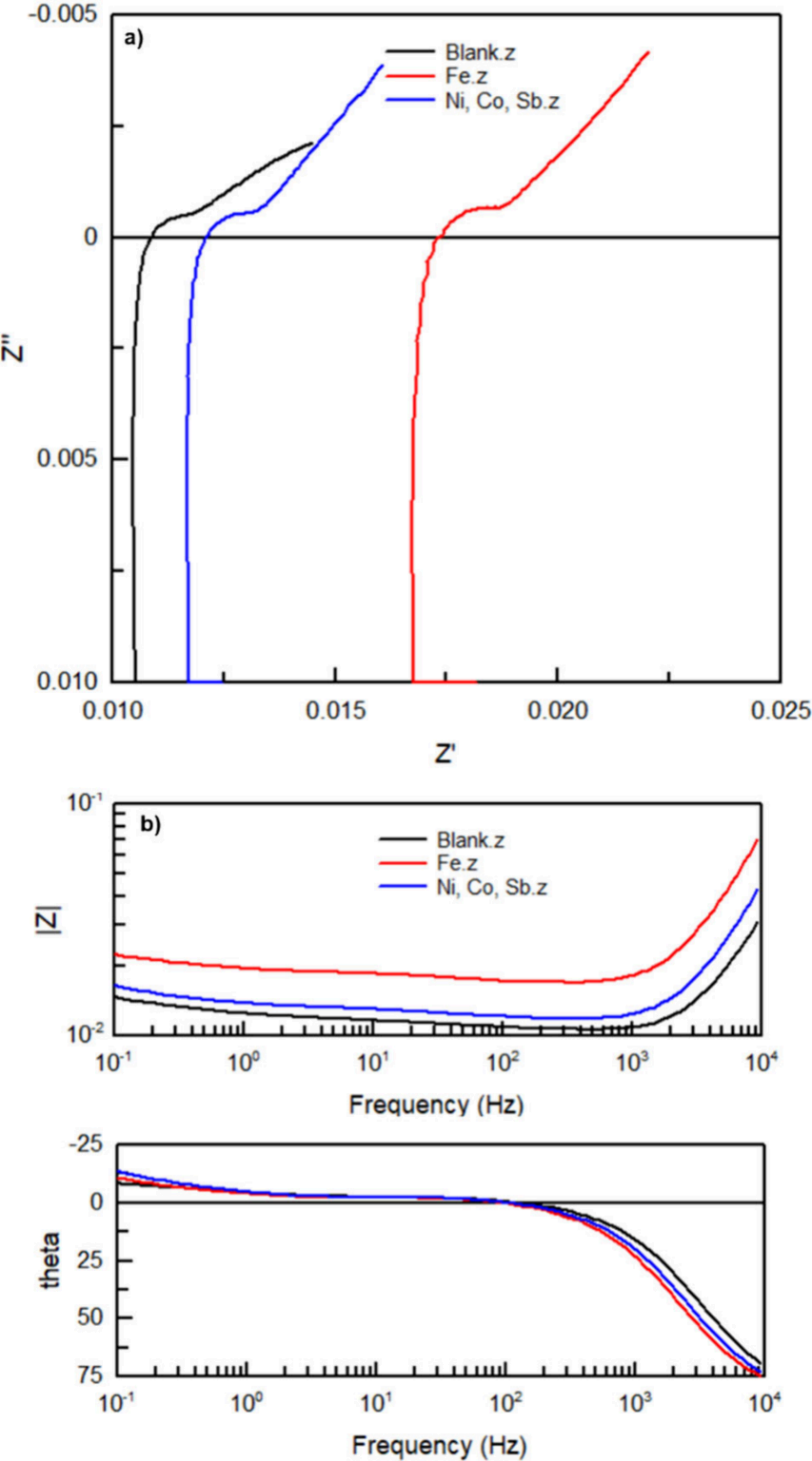
Nyquist plots (a) and Bode plots (b) obtained from EIS of a battery electrolyzer cell without additive (black), with Ni:Co:Sb 66:17:17 ppm additive (blue), and with 250 ppm iron additive (red).

The insights gained from EIS were required for understanding electrochemical processes, identifying internal resistance, detecting degradation, ensuring quality control during manufacturing, and monitoring the health of the battery over time. EIS provided valuable information about cell performance and efficiency. The Nyquist plot and a Bode plot obtained from a battery electrolyzer cell without additive, with Ni:Co:Sb 66:17:17 and 250 ppm Fe additive are displayed in Figure 18.

The Nyquist plot obtained from electrical impedance spectroscopy (EIS) of the lead-acid battery exhibits the expected characteristics for standard lead acid battery operation, including the semicircular shape that is useful for analyzing the charge transfer resistance at the electrode–electrolyte interface. The diameter of this semicircle directly correlates with the charge transfer resistance, with a larger diameter indicating higher resistance. Additionally, the low-frequency region of the Nyquist plot, which forms a straight line at approximately 45°, represents the diffusion-controlled process of ion transport within the battery. This observed behavior aligns with the typical impedance response of lead-acid batteries and is consistent with the characteristics reported by Hoppecke, confirming that the electrochemical and transport processes within the battery are operating as expected.^4,26−28^ The conformity of these results suggests that the battery has undergone effective manufacturing and possesses impedance features that match industry standards for lead-acid technology despite the modifications to the cell.

In the modified electrolyte containing Ni, Co, and Sb (66:17:17 ppm), the Nyquist plot shows a slight shift to higher real impedance (Z’) values, indicating a marginal increase in charge transfer resistance. However, this increase remains small (>0.01), with both Nyquist and Bode plots remaining similar to the baseline, confirming that the additives have not significantly impacted the internal resistance or overall electrochemical performance of the cells. Upon the introduction of iron (250 ppm) into the electrolyte, a further shift to slightly higher Z’ values (0.011 to 0.018) is observed, indicating an increase in internal resistance. Despite this shift, the overall impedance changes are small, and the cells continue to operate efficiently.

It should be noted that the values obtained from these EIS measurements are slightly higher than those reported for Hoppecke batteries, likely due to the increased electrolyte path caused by the addition of the gas separator. However, the additional losses incurred by this increased path length are considered acceptable at this stage, and the results confirm that the cell operates within acceptable performance limits. Overall, the small changes in impedance across all electrolyte modifications suggest that the internal resistance of the cells remains well-managed and the battery maintains efficient electrochemical functionality.

### Gas Chromatography Results

To test the purity of the gas, a sample of the hydrogen and oxygen were collected and passed through a gas chromatograph (chromatographs displayed in Supporting Information, Figure S3 and Figure S4). The carrier gas for testing the gas collected on the oxygen side was Helium, while that for the gas collected from the hydrogen terminal was Argon.

The results obtained demonstrated a high degree of purity >99% with no detectable crossover between gases. This is important to ensure that the hydrogen level is maintained below the LEL in oxygen and above the HEL on the hydrogen side.^25^

Additionally, GC analysis of the sulfur impurities present in the hydrogen sample collected from the 20-cell string of battery electrolyzer cells connected in series showed a concentration of 0.221 ppm hydrogen sulfide (H_2_S) when electrolyzing at 2.9 V per cell (56 V for 20 cells) and a current of 9.1 A. This level of H_2_S was below the limit of detection and is a sufficiently low concentration to ensure a high purity of hydrogen gas. At higher power, when electrolyzing using a voltage of 3.1 V and a current of 20 A, the measured concentration of H_2_S increased to 0.443 ppm. Therefore, operating at a lower power improves the purity of the hydrogen gas obtained from a string of battery electrolyzer cells.

## Conclusion

A low-cost method of green hydrogen production was successfully achieved using lead acid battery technology. The redesign of the cell allowed pure hydrogen to be collected from the NAM of the cell during electrolysis. Various metal additives improved the hydrogen yield obtained from the battery electrolyzer cell.

Among the additives tested, 100 ppm nickel sulfate was highly effective as a catalytic additive, significantly shifting the hydrogen evolution curve to a less negative voltage (−1.65 to −1.54 V), indicating its ability to facilitate hydrogen generation. Nickel showed no effect on the battery’s performance of the battery. In contrast, the addition of manganese sulfate did not exhibit discernible effects on the hydrogen evolution process, while cobalt demonstrated some catalytic behavior, by shifting the end voltage from −1.62 to −1.53 V. Antimony sulfate, although displaying a more subdued catalytic effect (−1.58 to −1.55 V), confirmed its compatibility with the lead plates, supporting its usage within the lead-acid combined battery and electrolysis system.

Although a combination of nickel, cobalt, and antimony resulted in a shift to a lower voltage of −1.50 V during cyclic voltammetry testing, no increase in hydrogen yield was achieved beyond that measured on addition of nickel. An increase of 75% hydrogen production rate was achieved on addition of 66 ppm of nickel additive to an unmodified 4 plate pair lead acid battery, while 50% increase was achieved on a modified battery electrolyzer with 1 plate pair. Therefore, it is confirmed that nickel is an attractive option for addition to the 4 M sulfuric acid electrolyte to increase hydrogen yield when the cell is electrolyzed using a voltage of 2.9 V and 9.1 A. At higher power, however, the catalytic effect becomes less significant.

Hydrogen gas produced from the battery electrolyzer cell operated at 2.9 V and 9.1 A had a high purity, with H_2_S recorded below the limit of detection by the gas chromatograph analysis. When electrolyzing at higher power, the impurity of H_2_S doubled but remained low. The low level of impurities measured confirmed that hydrogen produced from a battery electrolyzer can be successfully used as a fuel source for clean cooking.

## Supporting Information Available

Gas chromatography results obtained from hydrogen and oxygen sample collection from the battery electrolyzer cell during electrolysis. The rate of hydrogen and oxygen obtained from electrolysis (PDF)

## Abbreviations

HER: hydrogen evolution reaction
DOE: design of experiments
TCD: thermal conductive detector
SCD: sulfur chemiluminescence detector
NAM: negative active mass
PAM: positive active mass
OER: oxygen evolution reaction
BCI: Battery Council International
GC: gas chromatography

## References

[ref1] McKeon B. B.; Furukawa J.; Fenstermacher S. Advanced Lead-Acid Batteries and the Development of Grid-Scale Energy Storage Systems. Proc. IEEE 2014, 102 (6), 951–963. 10.1109/JPROC.2014.2316823.

[ref2] Kebede A. A.; Coosemans T.; Messagie M.; Jemal T.; Behabtu H. A.; Van Mierlo J.; Berecibar M. Techno-Economic Analysis of Lithium-Ion and Lead-Acid Batteries in Stationary Energy Storage Application. J. Energy Storage 2021, 40 (June), 102748 10.1016/j.est.2021.102748.

[ref3] May G. J.; Davidson A.; Monahov B. Lead Batteries for Utility Energy Storage: A Review. J. Energy Storage 2018, 15, 145–157. 10.1016/j.est.2017.11.008.

[ref4] Pavlov D. Invention and Development of the Lead–Acid Battery. Lead-Acid Batteries: Science and Technology 2011, 3–32. 10.1016/b978-0-444-59552-2.00001-8.

[ref5] Trasatti S. Work Function, Electronegativity, and Electrochemical Behaviour of Metals. III. Electrolytic Hydrogen Evolution in Acid Solutions. J. Electroanal. Chem. 1972, 39 (1), 163–184. 10.1016/S0022-0728(72)80485-6.

[ref6] Jenkins B.; Squires D.; Barton J.; Strickland D.; Wijayantha K. G. U.; Carroll J.; Wilson J.; Brenton M.; Thomson M. Techno-Economic Analysis of Low Carbon Hydrogen Production from Offshore Wind Using Battolyser Technology. Energies 2022, 15 (16), 5796 10.3390/en15165796.

[ref7] Lin Z.; Li K.; Tong Y.; Wu W.; Cheng X.; Wang H.; Chen P.; Diao P. Engineering Coupled NiSx-WO2.9 Heterostructure as PH-Universal Electrocatalyst for Hydrogen Evolution Reaction. ChemSusChem 2023, 16 (2), 1–9. 10.1002/cssc.202201985. 36394154

[ref8] Tong Y.; Chen P.; Chen L.; Cui X. Dual Vacancies Confined in Nickel Phosphosulfide Nanosheets Enabling Robust Overall Water Splitting. ChemSusChem 2021, 14 (12), 2576–2584. 10.1002/cssc.202100720. 33880883

[ref9] Tong Y.; Sun Q.; Chen P.; Chen L.; Fei Z.; Dyson P. J. Nitrogen-Incorporated Cobalt Sulfide/Graphene Hybrid Catalysts for Overall Water Splitting. ChemSusChem 2020, 13 (18), 5112–5118. 10.1002/cssc.202001413. 32672900

[ref10] Barton J.; Brenton M.; Abdullahi A.; Wilson J.; Ashton E.; Isherwood P.; Strickland D.; Thomson M.; Wijayantha U. Investigation of Different Acidic Battolyser Conditions for Energy Storage and Hydrogen Production. 58th Int. Univ. Power Eng. Conf. UPEC 2023 2023, 1–6. 10.1109/UPEC57427.2023.10294380.

[ref11] Besserguenev A. V.; Fogg A. M.; Francis R. J.; Price S. J.; O’Hare D.; Isupov V. P.; Tolochko B. P. Synthesis and Structure of the Gibbsite Intercalation Compounds [LiAl 2 (OH) 6]X {X = Cl, Br, NO 3} and [LiAl 2 (OH) 6]Cl·H 2 O Using Synchrotron X-Ray and Neutron Powder Diffraction. Chem. Mater. 1997, 9 (1), 241–247. 10.1021/cm960316z.

[ref12] Wang S.; Lu A.; Zhong C. J. Hydrogen Production from Water Electrolysis: Role of Catalysts. Nano Converg. 2021, 8 ( (1), ). 10.1186/s40580-021-00254-x. PMC7878665 33575919

[ref13] Barton J. P.; Gammon R. J. L.; Rahil A. Characterisation of a Nickel-Iron Battolyser, an Integrated Battery and Electrolyser. Front. Energy Res. 2020, 8, 1–15. 10.3389/fenrg.2020.509052.

[ref14] Ballantyne A. D.; Hallett J. P.; Riley D. J.; Shah N.; Payne D. J. Lead Acid Battery Recycling for the Twenty-First Century. R. Soc. Open Sci. 2018, 5 (5), 171368 10.1098/rsos.171368. 29892351 PMC5990833

[ref15] Pavlov D.; Naidenov V.; Ruevski S. Influence of H2SO4 Concentration on Lead-Acid Battery Performance. H-Type and P-Type Batteries. J. Power Sources 2006, 161 (1), 658–665. 10.1016/j.jpowsour.2006.03.081.

[ref16] Dadallagei K. S. R.; Parr IV D. L.; Coduto J. R.; Lazicki A.; DeBie S.; Haas C. D.; Leddy J. Perspective—New Perspectives from Classical Transition State Theory: The Hydrogen Evolution Reaction on Metal Electrodes. J. Electrochem. Soc. 2023, 170 (8), 086508 10.1149/1945-7111/acf246.

[ref17] Technical Manual BCIS-03A: Recommended Test Methods for VRLA-AGM Separators https://batterycouncil.org/resource/technical-manual-bcis-03a-recommended-test-methods-for-vrla-agm-separators/ (accessed Jun 12, 2024).

[ref18] Pierson J. R.; E Weinlein C.; E Wright c. Determination of acceptable contaminant ion concentration levels in a truly maintenance-free lead acid battery. Power Sources 5: Research and Development in Non-mechanical Electrical Power Sources: Proceedings of the 9th International Symposium Held at Brighton 1974, 5, 97.

[ref19] Ashton E.; Oakley W. C.; Brack P.; Dann S. E. Evaluation of the Vapor Hydrolysis of Lithium Aluminum Hydride for Mobile Fuel Cell Applications. ACS Appl. Energy Mater. 2022, 5, 8336 10.1021/acsaem.2c00891. 35909805 PMC9326813

[ref20] Pavlov D.; Dakhouche A.; Rogachev T. Influence of Antimony on the Electrochemical Behaviour and the Structure of the Lead Dioxide Active Mass of Lead/Acid Batteries. J. Power Sources 1993, 42 (1–2), 71–88. 10.1016/0378-7753(93)80138-F.

[ref21] Gennero de Chialvo M. R.; Chialvo A. C. Hydrogen Evolution Reaction%: Analysis of the Volmer-Heyrovsky-Tafel Mechanism with a Generalized Adsorption Model. J. Electroanal. Chem. 1994, 372, 209–223.

[ref22] Murthy A. P.; Theerthagiri J.; Madhavan J. Insights on Tafel Constant in the Analysis of Hydrogen Evolution Reaction. J. Phys. Chem. C 2018, 122 (42), 23943–23949. 10.1021/acs.jpcc.8b07763.

[ref23] Vinal G. W.; Craig D. N.; Snyder C. L. Note on the Effects of Cobalt and Nickel in Storage Batteries. J. Res. Natl. Bur. Stand. (1934). 1940, 25, 417–420. 10.6028/jres.025.018.

[ref24] Visscher W. Cyclic Voltammetry on Lead Electrodes in Sulphuric Acid Solution. J. Power Sources 1976, 1 (3), 257–266. 10.1016/0378-7753(76)81003-8.

[ref25] Romero A. F.; Urra O.; Blecua M.; Ocón P.; Valenciano J.; Trinidad F. Effect on Water Consumption by Metallic Impurities into Electrolyte of Lead-Acid Batteries. J. Energy Storage 2021, 42, 103025 10.1016/j.est.2021.103025.

[ref26] HOPPECKE Batterien GmbH & Co. KG. Installation, commissioning and operating instructions, for vented stationary lead-acid batteries https://www.hoppecke.com/fileadmin/Redakteur/Hoppecke-Main/Products/Downloads/Montagehandbuch_geschl_EN_final.pdf (accessed Nov 21, 2024).

[ref27] Mohsin M.; Picot A.; Maussion P. A New Lead-Acid Battery State-of-Health Evaluation Method Using Electrochemical Impedance Spectroscopy for Second Life in Rural Electrification Systems. J. Energy Storage 2022, 52 (PA), 104647 10.1016/j.est.2022.104647.

[ref28] Majchrzycki W.; Jankowska E.; Baraniak M.; Handzlik P.; Samborski R. Electrochemical Impedance Spectroscopy and Determination of the Internal Resistance as a Way to Estimate Lead-Acid Batteries Condition. Batteries 2018, 4 (4), 70 10.3390/batteries4040070.

