# Supplementary material for: Evaluation of the Catalytic Effect of Metal Additives on the Performance of a Combined Battery and Electrolyzer System
Source: ACS Appl Energy Mater. 2025 Jan 8;8(2):1112–25. doi: 10.1021/acsaem.4c02648 (PMC11776374; doi:10.1021/acsaem.4c02648)
Supplement: Supplementary file 1 — ae4c02648_si_001.pdf [file ae4c02648_si_001.pdf]

Supporting Information

E. Ashton[1]*, M. Brenton[1], J. G. Wilson[1], J. P. Barton[1], R. Wilson[1], D. Strickland[1], S. A. Kondrat[2], N. Clement[3], J. Wertz[3], J. Zhang[3]

[1] CREST, Wolfson School of Mechanical, Electrical and Manufacturing Engineering, Loughborough University, Loughborough, LE11 3TU, UK

[2] Department of Chemistry, School of Science, Loughborough University, Loughborough, LE11 3TU, UK

[3] Hollingsworth and Vose, Groton, MA 01450, USA

* e.ashton@lboro.ac.uk

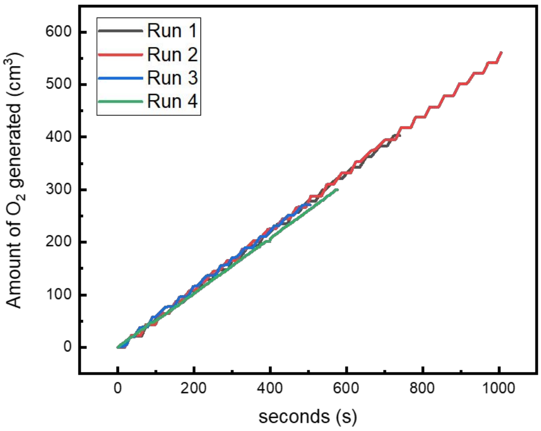

FIGURE S1 MEASURED OXYGEN COLLECTION RATES OVER A NUMBER OF TEST RUNS

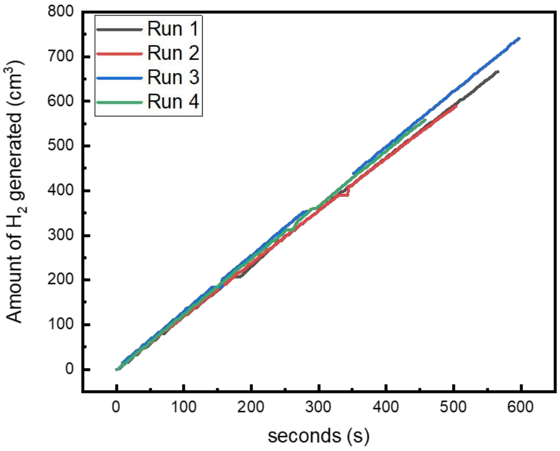

FIGURE S2 MEASURED HYDROGEN COLLECTION RATES OVER A NUMBER OF TEST RUNS.

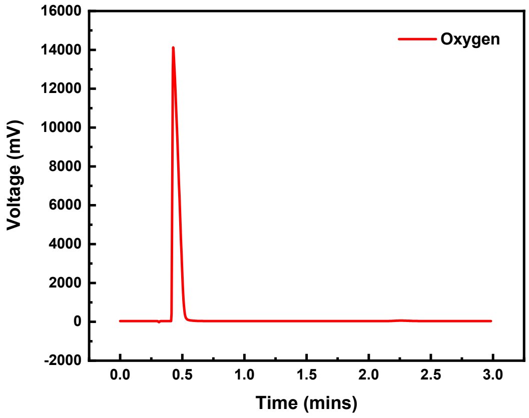

FIGURE S3 GC RESULT FOR GAS COLLECTED FORM THE OXYGEN TERMINAL.

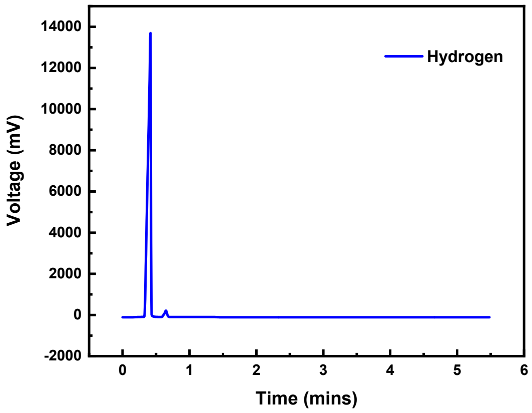

FIGURE S4 GC RESULT FOR GAS COLLECTED FORM THE HYDROGEN TERMINAL.
